# Supplementary material for: Rapid Expansion of Immune-Related Gene Families in the House Fly, Musca domestica
Source: Mol Biol Evol. 2017 Jan 12;34(4):857–72. doi: 10.1093/molbev/msw285 (PMC5400391; doi:10.1093/molbev/msw285)
Supplement: Supplementary Data [file msw285_Supp.zip › TABLES5.docx]

Table S5.

|  | Model Term | Effect Size | Standard Error | P-value |
| --- | --- | --- | --- | --- |
| response variable: turnover  rate | (Intercept) | -5.917 | 0.01677 | < 2e-16 |
|  | *Musca* branch = TRUE | 0.253 | 0.02539 | < 2e-16 |
|  | Immune gene = TRUE | 0.529 | 0.08583 | 7.1e-10 |
|  | *Musca* x Immune Interaction | 0.130 | 0.07571 | 0.0867 |
| response variable: duplication  rate | (Intercept) | -6.393 | 0.01929 | < 2e-16 |
|  | *Musca* branch = TRUE | 0.631 | 0.02827 | < 2e-16 |
|  | Immune gene = TRUE | 0.542 | 0.09335 | 6.32e-09 |
|  | *Musca* x Immune Interaction | 0.314 | 0.08325 | 0.00016 |
| response  variable:  loss rate | (Intercept) | -7.419 | 0.03347 | < 2e-16 |
|  | *Musca* branch = TRUE | -0.902 | 0.07134 | < 2e-16 |
|  | Immune gene = TRUE | 0.644 | 0.14262 | 6.29e-06 |
|  | *Musca* x Immune Interaction | -0.257 | 0.22640 | 0.256 |
